# Konzo prevention in six villages in the DRC and the dependence of konzo prevalence on cyanide intake and malnutrition

**DOI:** 10.1016/j.toxrep.2015.03.014

**Published:** 2015-04-07

**Authors:** J.P. Banea, J. Howard Bradbury, C. Mandombi, D. Nahimana, Ian C. Denton, Matthew P. Foster, N. Kuwa, D. Tshala Katumbay

**Affiliations:** a Programme National de Nutrition (PRONANUT), Kinshasa, The Democratic Republic of the Congo; b EEG, Research School of Biology, Australian National University, Canberra, ACT 0200, Australia; c Hopital General de Reference, Zone de Sante de Popokabaka, The Democratic Republic of the Congo; d Centre Neuropsychopathologique, Universite de Kinshasa, The Democratic Republic of the Congo

**Keywords:** Konzo prevalence, Urinary thiocyanate, Malnutrition, Konzo prevention, Wetting method, Cassava cyanide

## Abstract

Six villages in Boko Health Zone, Bandundu Province, DRC, were studied with 4588 people, 144 konzo cases and konzo prevalences of 2.0–5.2%. Konzo incidence is increasing rapidly in this area. Food consumption scores were obtained from the households with konzo and the mean % malnutrition calculated for each village. Urine samples were obtained from 50 school children from each village and % high urinary thiocyanate content (>350 μmol/L) determined. The experimental data relating % konzo prevalence (%K) to % children with high urinary thiocyanate content (%T) and % malnutrition (%M) for the six villages were fitted to an equation %K = 0.06%T + 0.035%M. This confirms that konzo is due to a combination of high cyanide intake and malnutrition. The village women used the wetting method to remove cyanogens from cassava flour. During the 9-month intervention there were no new cases of konzo; cyanide in flour had reduced to WHO safe levels and mean urinary thiocyanate levels were greatly reduced. To prevent konzo at least 60–70% of women should use the wetting method regularly. The wetting method is now accepted by the World Bank, FAO and WHO as a sensitive intervention. Four successful konzo interventions have involved nearly 10,000 people in 13 villages, the cost is now $16 per person and the methodology is well established.

## Highlights

• In 6 villages we measured konzo prevalence, urinary thiocyanate and FC scores.
• % konzo prevalence (%K), % high urine thiocyanate (%T), % malnutrition (%M) relate.
• The results fitted an equation %K = 0.06%T + 0.035%M.
• The wetting method was used by women over a 9-month intervention to prevent konzo.
• The methodology has now been used with nearly 10,000 people in 13 villages.

## 1 Introduction

Konzo is an upper motor neuron disease that causes irreversible paralysis of the legs particularly in children and young women and is very likely due to high cyanogen intake amongst people with malnutrition, who consume a monotonous diet of bitter cassava [1], [2], [3], [4]. Konzo occurs in the Democratic Republic of Congo (DRC), Mozambique, Tanzania, Cameroon, Central African Republic, Angola and possibly Congo [5], [6]. It has been found that the % mean monthly incidence of konzo across the year is significantly related to the monthly % of children with high urinary thiocyanate levels, which is a measure of cyanogen intake [4]. For example, there is a high incidence of konzo cases during the dry season when cassava is harvested and urinary thiocyanate levels are high and conversely low konzo incidence in the wet season when consumption of cassava and urinary thiocyanate levels is low.

That adequate nutrition may prevent konzo was shown in three unrelated konzo outbreaks in Mozambique, DRC and Tanzania, in which people of the same ethnic group living only 5 km from those with high konzo prevalence had a konzo prevalence close to zero. Those with near zero konzo prevalence in Mozambique lived near the sea [7], in DRC they lived in the forest and urban centres [8] and in Tanzania they lived close to Lake Victoria [9]. These people had a much better protein intake due to availability of fish from the sea and from Lake Victoria and animals from the forest. The animal protein that protected them from contracting konzo had an adequate supply of the sulphur amino acids methionine and cystine/cysteine that are required to detoxify cyanide to thiocyanate in the body. In a recent study Okitundu et al. [10] showed that chronic cyanide intoxication, malnutrition, poverty and superstitious beliefs all favoured the persistence of konzo in Kahemba Health Zone, Bandundu Province, DRC.

Konzo has previously been prevented in three studies in seven villages in Popokabaka and Boko Health Zones in Bandundu Province, DRC, by education of the village women to use the wetting method every day to remove cyanogens from cassava flour [4], [6], [11]. In this paper we have data on the konzo prevalence, malnutrition and high cyanide intake in six villages and have developed a simple mathematical relationship between them. The wetting method has been used to prevent konzo in these villages.

## 2 Materials and methods

### 2.1 Study area

**Fig. 1:**
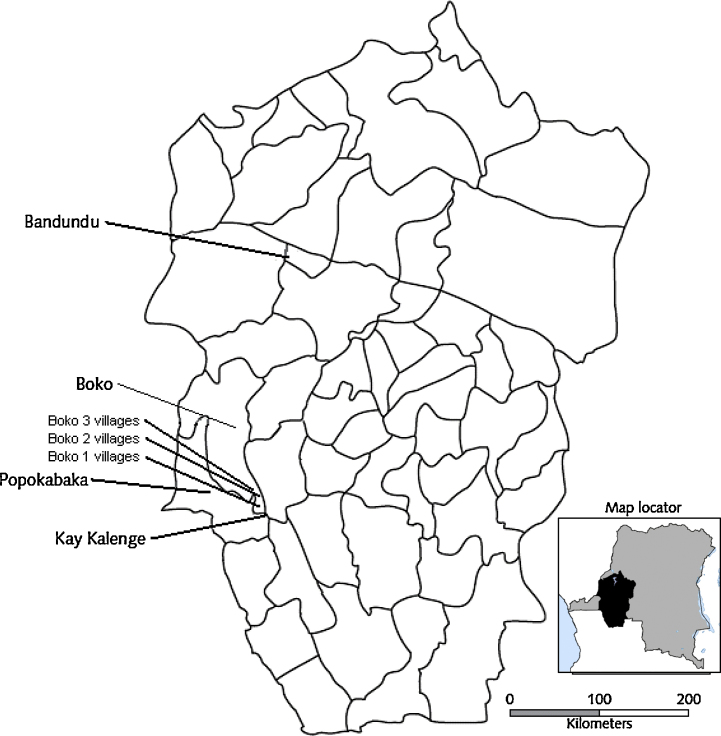
The health zones of Bandundu Province including Boko and Popokabaka health zones (marked) and the locations of the konzo intervention in six Boko 3 villages (this paper), three Boko 2 villages [4], three Boko 1 villages [11] and the first intervention in Kay Kalenge village [6].

After initial discussions with the Kwango District medical officer and the coordinator of nutrition in Kenge City, it was decided to work in the Boko Health Zone where there were villages badly affected by konzo, and also that all personnel in Boko Health Zone should receive training in use of the wetting method. Six villages were chosen from three health areas (Kitati, Mutombo and Tsakela Mbewa) based on the prevalence of konzo, the presence of capable village leaders and accessibility by car. The location of the six villages is shown in Fig. 1, where they are labelled Boko 3 villages. Makiku, Kitati and Kipesi villages are in the forest and Mangungu, Mutombo and Kinkamba villages are in the savannah. Each village has a primary school, there is a health centre in Mutombo and a health post in Makiku. Clean drinking water was installed 4 years ago by UNICEF in Mutombo, but the small rivers in the area do not provide clean drinking water for the other villages.

### 2.2 Agriculture and processing

Bitter cassava is grown almost exclusively because sweet cassava is often stolen. New, less bitter cassava varieties were introduced by FAO into Mutombo, Mangungu and Kinkamba. The fallow period has been reduced to 3 year from 7 to 10 years, which was normal some years ago. Cassava, maize, cowpea, taro, squash, plantain, okra, pineapple, tomato and yam are grown and there are goats, pigs and chickens. Cassava is processed by soaking (retting) in water generally for 2 days and dried for 1–4 days depending on the weather. There is trade in cassava during the dry season and corn during corn harvest.

### 2.3 Second visit to the six villages

The second visit in September 2013 involved the full team of workers and a population census was carried out. Suspected konzo cases were examined by two medical doctors following a standardised WHO protocol for konzo as follows: (1) a spastic visible walk or run, (2) a history of sudden onset within a week of a formerly healthy person, (3) exaggerated bilateral patellar or achillian reflexes and (4) non-progressive evolution of the disease [12]. The month and year of onset of konzo was recorded for each konzo case. Konzo sufferers were given multivitamins and anti-inflammatory drugs. A socio-economic and food consumption survey was conducted among 138 households with at least one konzo case. It was ascertained how many meals were eaten the day before the survey and the number of days of different foods eaten during the week preceding the survey. The food consumption score (FCS) was calculated and interpreted using the methodology of the World Food Program [13], [14]. From each village, 30 cassava flour samples were obtained and 50 urine samples from school children with verbal agreement of their parents. The samples were analysed on site with kits supplied from Australia, using methods described below.

The wetting method for removing cyanogens from cassava flour was taught first to 10–12 of the leading women of the village. Each trained woman then trained 10–15 others in the village. In each village a committee was formed to ensure follow up of the training. After the training each woman was given a plastic basin, a knife and a mat. Using the wetting method cassava flour was placed in a plastic basin and a mark made on the inside of the bowl with a knife. Water was added with mixing and the volume decreased and then increased up to the mark. The wet flour was spread out in a layer not more than 1 cm thick and stood for 2 h in the sun or 5 h in the shade, to allow hydrogen cyanide gas to escape to the air [15], [16], [17]. The damp flour was then cooked in boiling water in the traditional way to make a thick porridge called fufu, which was eaten with pounded, boiled cassava leaves or another food to give it flavour.

### 2.4 Subsequent visits to the six villages

Each month after the second visit, the Caritas Mandombi team visited the villages to check on the use of the wetting method until March 2014 when there was a third visit of the full team. Using non-structured interviews with village leaders and women, a check was made for any new konzo cases and on the continued use of the wetting method. The daily use of the wetting method by each family was recorded, from which the percentage of families using the wetting method was calculated. Also 30 cassava flour samples, which had already been treated using the wetting method, were collected from each village and 50 urine samples obtained from school children. Monthly visits of the Caritas Mandombi team continued until July 2014, when the fourth and final visit by the full team was made. Using focus groups, checks were made in each village for any new cases of konzo and on the number of women using the wetting method. As before, about 30 flour samples treated by the wetting method were collected from each village and 50 urine samples from school children for analysis on site.

### 2.5 Urinary thiocyanate analysis

Fifty urine samples were collected randomly from school age children in each village with oral consent of their parents and a record made of their age and sex. These samples were analysed on site using the simple picrate thiocyanate kit D1 ([18]; http://biology.anu.edu.au/hosted_sites/CCDN/). A colour chart with 10 shades of colour from yellow to brown was used, which corresponded to 0–1720 μmol thiocyanate/L.

### 2.6 Total cyanide analysis

Thirty samples of cassava flour about to be used to prepare fufu were collected randomly from households in each village before teaching the wetting method in September 2013 and at subsequent visits in March and July 2014. Analyses for total cyanide were made using a simple picrate kit B2 ([19], [20]; http://biology.anu.edu.au/hosted_sites/CCDN/). A colour chart with 10 shades of colour from yellow to brown was used, which corresponded to 0–800 mg HCN equivalents/kg cassava flour = ppm.

### 2.7 Relation among konzo prevalence, high urinary thiocyanate and malnutrition

Percentage konzo prevalence (%K) was considered to be a function of both cyanide intake, measured by % of children with a high urinary thiocyanate content (%T) and % malnutrition (%M). The %M was calculated from the experimental data in Table 2 by weighting the % of konzo families with a poor food consumption score (FCS) and adding to it the value of the % of konzo families with a limited FCS, using the equation

(1) $$\%\;\text{M}=0.5\left[2\left(\text{poor}\;\text{FCS}\right)+\text{limited}\;\text{FCS}\right]$$

Two types of relation between konzo prevalence (%K), % high urinary thiocyanate content (%T) and % malnutrition (%M) were considered.

(a) An additive relationship in which konzo prevalence (%K) was the sum of two terms based on %T and %M, as follows:
  (2) %K=x %T+y %M.
(b) A multiplicative relation in which %T was multiplied by %M,
  (3) $$\%\text{K}=z\left(\%\text{T}\right)\left(\%\text{M}\right)\text{.}$$

The best values for *x*, *y* and *z* in Eqs. (2), (3) were obtained by iteration using the data for six villages of %K, %T and %M (see Table 1, Table 2, Table 3).

**Table 1:** Population, number of konzo cases and % konzo prevalence in six Boko villages.

Village	Population	Number of konzo cases	% Konzo prevalence (%K)
Makiku	1324	38	2.8
Kitati	863	24	2.8
Kipesi	646	26	4.0
Mangungu	721	23	3.2
Mutombo	653	13	2.0
Kinkamba	381	20	5.2
Total	4588	144	3.1

**Table 2:** % of konzo households with poor, limited and acceptable food consumption scores (FCS) and % malnutrition (%M).

Village	Percentage of konzo households with			
	Poor FCS^a^	Limited FCS^b^	Acceptable FCS^c^	Malnutrition (%M)^d^
Makiku	49	34	17	66
Kitati	14	32	54	30
Kipesi	88	12	0	94
Mangungu	5	95	0	53
Mutombo	54	31	15	70
Kinkamba	94	6	0	97
Mean	51	35	14	69

a FCS < 28

b FCS = 28–42

c FCS > 42

d % malnutrition = 0.5 [2(poor FCS) + (limited FCS)]

**Table 3:** Urinary thiocyanate and mean cassava flour cyanide levels in six konzo villages, before teaching the wetting method.

Village	% of children with high urinary thiocyanate (%T)^a^	Mean urinary thiocyanate (μmol/L)^b^	Mean total cyanide of cassava flour (ppm)^c^
Makiku	28	427	20
Kitati	22	335	22
Kipesi	20	315	19
Mangungu	31	530	20
Mutombo	48	729	41
Kinkamba	17	330	41

a % of children with urinary thiocyanate content > 350 μmol/L.

b Mean of 50 urine samples obtained with verbal assent of parents.

c Mean of 30 cassava flour samples from each village.

## 3 Results

**Fig. 2:**
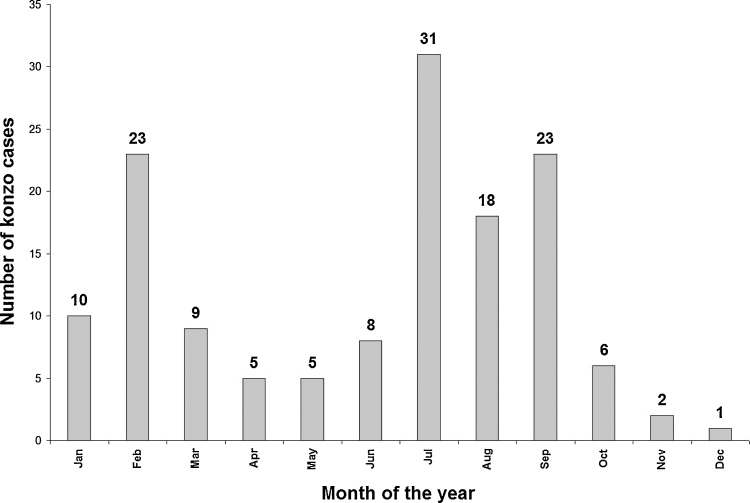
Monthly distribution of onset of konzo in the six Boko villages.

**Fig. 3:**
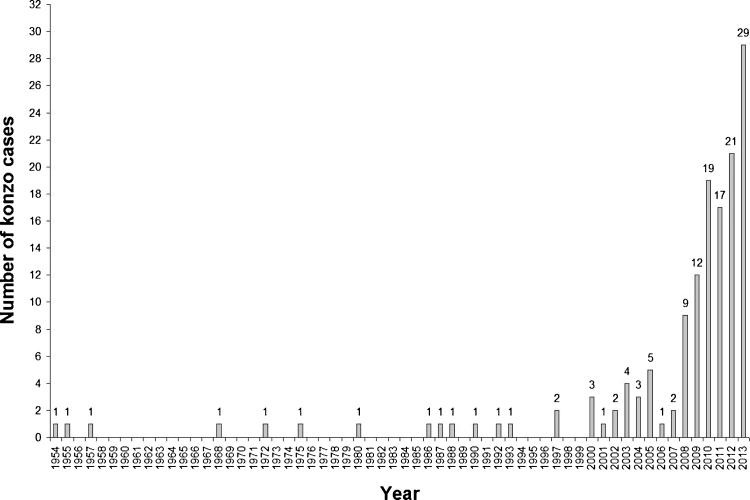
Annual distribution of onset of konzo cases in six villages stretching back over 59 years, with incidence every year since 2000.

There was a total of 144 konzo cases in the six villages, with a mean konzo prevalence of 3.1% (see Table 1). Konzo onset usually occurred in the morning. There were 5% severely disabled who could not walk, 27% moderately disabled who needed one or two sticks and 68% mildly disabled who did not need a stick. Impaired vision was found in 40% and speech disorders in 13% of cases. Nearly half of the patients had a family member with konzo. All konzo households lived in poverty in straw houses which leaked water in the rainy season and 31% said they were living on donations from others. Only 28% had a radio and 7% a bicycle. Fig. 2, Fig. 3 showed the month and the year of occurrence of the konzo cases respectively.

### 3.1 Food consumption and % malnutrition in konzo households

The food survey showed that the day before the survey, there were 1% of konzo households who had not eaten anything, 50% had one meal, 19% had two meals and 30% had three or more meals. The mean number of days that food was consumed per week by konzo families was cassava flour 7.0, vegetables which included cassava leaves 6.0, collection products (roots, caterpillars) 5.0, cooking oil 2.0, fruit 1.6, meat and fish 1.6, sugar 1.3, cereals 1.2, legumes 0.8 and milk 0.8. The food consumption score (FCS) for the households with konzo were calculated and the averaged values for each village are shown in Table 2 along with the calculated value of the % malnutrition [13], [14].

### 3.2 Dependence of % konzo prevalence on % children with high urinary thiocyanate and % malnutrition

For the six villages the % children with urinary thiocyanate levels >350 μmol/L, before teaching the wetting method, are shown in Table 3. This data, together with that for % malnutrition (Table 2), were substituted in Eqs. (2), (3) and the best fit values of *x*, *y* and *z* were obtained by iteration. It was found that Eq. (2) fitted the data better than Eq. (3), using values for *x* and *y* of 0.06 and 0.035, respectively. Substituting in Eq. (2) gives

(4) % K=0.06% T+0.035% M.

Using the values of %T and %M from Table 3, Table 2 respectively, the following values were found for the calculated %K in the six villages, with the actual value shown in brackets: Kinkamba 4.4 (5.2), Kipesi 4.5 (4.0), Mangungu 3.7 (3.2), Makiku 4.0 (2.8), Kikati 2.4 (2.8) and Mutombo 5.3 (2.0).

### 3.3 Prevention of konzo by women using the wetting method

Table 4 shows the mean urinary thiocyanate content of school children over the intervention and also the % of families who regularly used the wetting method in July 2014. Table 5 gives the % of school children with high urinary thiocyanate content (>350 μmol/L) over the time of the intervention. In the six villages the mean total cyanide content of cassava flour measured just before it was used to make fufu reduced from the value of 19 to 41 ppm before the wetting method was taught (see Table 3), to the following in July 2014: Makiku 9 ppm, Kitati 9 ppm, Kipesi 8 ppm, Mangungu 12 ppm, Mutombo 11 ppm and Kinkamba 8 ppm.

**Table 4:** Mean thiocyanate content (μmol/L) of urine of school children^a^ and % use of wetting method by families in six villages.

Village	Mean urinary thiocyanate content^a^ in μmol/L			% of families using
	September 2013^b^	March 2014	July 2014	Wetting method^c^
Makiku	427	173	184	74
Kitati	335	244	165	78
Kipesi	315	207	179	83
Mangungu	530	242	160	94
Mutombo	729	411	280	68
Kinkamba	330	274	175	88
Mean	444	259	191	81

a Mean values from 60 samples.

b Results obtained before introduction of wetting method.

c % of families using the wetting method regularly in July 2014.

**Table 5:** Percentage of school children in six villages with high urinary thiocyanate content (>350 μmol/L).

Village	Percentage of children with urinary thiocyanate content of ≥350 μmol/L		
	September 2013^a^	March 2014	July 2014
Makiku	28	2	0
Kitati	22	11	5
Kipesi	20	7	2
Mangungu	31	10	0
Mutombo	48	34	8
Kinkamba	17	16	2

a Before introducing the wetting method.

## 4 Discussion

### 4.1 Relationship among konzo prevalence, high cyanide intake and malnutrition

Eq. (4) was developed by iteration from the data of six villages to relate the % konzo prevalence with the % children with high urinary thiocyanate content (%T) and % malnutrition (%M). There is a reasonable fit of the data for five villages, but Mutombo has a much lower actual konzo prevalence (2.0) than that expected from the equation (5.3). This is probably because Mutombo is the only village that has a health centre and a secure water supply. Both of these factors would improve the health of the Mutombo people and could have decreased their konzo prevalence. Since konzo occurs only when high cyanide intake and malnutrition occur together, such as occurs in remote villages particularly during drought or war [1], [5], Eq. (4) is inapplicable if either %T or %M is zero.

This relationship represents a first attempt to relate mathematically konzo prevalence with high cyanide intake and malnutrition and as shown by the result for Mutombo (and to a lesser extent for Makiku which has a health post) there are other health factors that bear on % konzo prevalence. However, Eq. (4) does greatly strengthen the long held hypothesis that konzo is associated with high cyanide intake and malnutrition from consumption of a monotonous diet of bitter cassava and malnutrition [1], [2], [4].

### 4.2 Konzo prevalence in six villages

There were 144 konzo cases in the six Boko 3 villages. Combining the data of those severely, moderately and mildly disabled (see results) with those from seven other studies in DRC and Tanzania, it is found that the mean percentage of konzo cases (standard deviations in brackets) severely disabled are 7 (4), moderately disabled 26 (12) and mildly disabled 67 (11) [4], [6], [9], [11], [14], [21], [22]. As shown in Fig. 2, konzo incidence peaks in July, the peak cassava season, with smaller peaks in February and September. Fig. 3 is particularly interesting for two reasons (1) the long span of years going back to 1954, before independence from Belgium, and (2) the sporadic incidence of konzo cases in earlier years compared with incidence every year over the last 14 years, rising to a very concerning maximum of 29 cases in 2013. We have noted with concern this large increase in konzo incidence in recent years in Popokabaka and Boko Health Zones of Kwango District [4], [6], [11] and in adjacent Kwilu District [14]. We believe that this is due to a decrease in production of cassava due to plant diseases and people being involved in other work. There is a socio-economic decline among the people, with many konzo families being supported by others (see Section 3).

### 4.3 Prevention of konzo by use of the wetting method

The teaching of the wetting method to the women and its subsequent daily use by them prevented the occurrence of any new cases of konzo and reduced the cyanide content of cassava flour to about 10 ppm, the maximum acceptable WHO level [23]. It also reduced the mean thiocyanate content of urine of school children in the villages (Table 4) and the % of children who had high urinary thiocyanate content (>350 μmol/L), see Table 5, and were in danger of developing konzo. The continuous reduction in urinary thiocyanate levels and in % of children in danger of contracting konzo was only achieved by good social mobilisation by the women, who formed a committee in each village and checked on the regular daily use of the wetting method by each household. This allowed collection of data on the percentage of families in each village who used the wetting method in July 2014. As shown in Table 4, there were 94% of families in Mangungu who used the wetting method which gave a mean urinary thiocyanate content of 160 μmol/L, whereas in Mutombo only 68% of families used the wetting method and the mean urinary thiocyanate content was much higher at 280 μmol/L (see also [11]). There is an inverse relation between the percentage of families who use the wetting method and the mean urinary thiocyanate content. The same effect is also reflected in Table 5, where there are 8% of school children with high urinary thiocyanate levels in Mutombo in July 2014 compared with 0% in Mangungu. For the wetting method to be effective in preventing konzo in a village, it is clear that at least 60–70% of women should be using the wetting method on a regular daily basis.

### 4.4 Comparison between prevention of konzo by reducing cyanide intake and by reducing malnutrition

We have now prevented konzo amongst nearly 10,000 people in 13 villages in Kwango District, DRC, by training the women to use the wetting method on cassava flour, as an additional processing method used before its consumption as fufu. The wetting method is popular with rural women and once established they continue to use it, and its use has spread by word of mouth to other nearby villages [24]. In the current work, the total cost of the intervention in six villages with 4588 people was US$ 75,000, which equals $16 per person. Another method of preventing konzo is to reduce malnutrition and a cross-sectoral approach has been used with some success by the NGO Action Against Hunger in Kwango District [25], [26]. Reducing the cyanogen intake using the wetting method appears to be a much more direct, effective and less expensive method of preventing konzo than by attempting to remove malnutrition, but a broader approach could see cyanide intake and malnutrition reduced together [27].

## 5 Conclusion

For six konzo villages the data for percentage konzo prevalence (%K), percentage malnutrition (%M) and percentage of children with high urinary thiocyanate levels (%T) are related by the equation

% K=0.06% T+0.035% M.

This equation fits the data fairly well, except for the village of Mutombo, which has a secure water supply and a health centre and hence its konzo prevalence is much lower than that calculated by the equation. The equation is a first attempt to relate mathematically konzo prevalence with high cyanide intake and malnutrition. It greatly strengthens the long held association between konzo incidence and high cyanide intake from a monotonous diet of bitter cassava that causes malnutrition [1], [2].

The wetting method has been recognised by the World Bank, WHO and FAO as a sensitive intervention to remove cyanogens from cassava flour. It should be promoted as an additional processing method to reduce the cyanide intake of the people in all tropical African countries, where cassava is being introduced into new areas in which there is no knowledge of the processing methods needed to remove cyanogens and cassava production is increasing to feed growing populations [5]. The wetting method has now been used successfully to prevent konzo in the DRC in 13 villages with nearly 10,000 people. The methodology used to prevent konzo is now well established and interventions require about 9 months and cost about $16 per person, which could be reduced further by scaling up the operation. Konzo is spreading geographically in tropical Africa [6], [28] and is intensifying in Bandundu Province (see Section 4.2). We appeal urgently to funding agencies worldwide for additional funding to tackle specifically the scourge of konzo and more broadly other cassava cyanide diseases in tropical Africa.

## Conflict of interest

The authors declare that there is no conflict of interest.

## Transparency document

Transparency document
